# Bioinformatics Analysis Reveals MicroRNAs Regulating Biological Pathways in Exercise-Induced Cardiac Physiological Hypertrophy

**DOI:** 10.1155/2017/2850659

**Published:** 2017-02-14

**Authors:** Jiahong Xu, Yang Liu, Yuan Xie, Cuimei Zhao, Hongbao Wang

**Affiliations:** ^1^Department of Cardiology, Tongji Hospital, Tongji University School of Medicine, Shanghai 200065, China; ^2^Department of Cardiology, Yangpu Hospital, Tongji University School of Medicine, Shanghai 200090, China

## Abstract

Exercise-induced physiological cardiac hypertrophy is generally considered to be a type of adaptive change after exercise training and is beneficial for cardiovascular diseases. This study aims at investigating exercise-regulated microRNAs (miRNAs) and their potential biological pathways. Here, we collected 23 miRNAs from 8 published studies. MirPath v.3 from the DIANA tools website was used to execute the analysis, and TargetScan was used to predict the target genes. Kyoto Encyclopedia of Genes and Genomes (KEGG) and Gene Ontology (GO) analyses were performed to identify potential pathways and functional annotations associated with exercise-induced physiological cardiac hypertrophy. Various miRNA targets and molecular pathways, such as Fatty acid elongation, Arrhythmogenic right ventricular cardiomyopathy (ARVC), and ECM-receptor interaction, were identified. This study could prompt the understanding of the regulatory mechanisms underlying exercise-induced physiological cardiac hypertrophy.

## 1. Introduction

Cardiac hypertrophy, including physiological hypertrophy and pathological hypertrophy, is one of the most important adaptive mechanisms for the heart in various situations of stress [1]. Pathological cardiac hypertrophy is always associated with decreased cardiac dysfunction and poor prognosis, which can ultimately lead to heart failure [2]. In contrast, physiological hypertrophy induced by pregnancy or chronic exercise training is favourable for cardiac function [2]. Various epidemiological and experimental studies revealed that exercise training contributes to physiological cardiac hypertrophy, and it can attenuate the pathological hypertrophy related to cardiovascular diseases, such as myocardial infarction, heart failure, and cardiomyopathy [3, 4].

The biological mechanism underlying the physiological cardiac hypertrophy induced by exercise is complex and still incompletely elucidated [3]. In recent years, an increasing number of studies have identified that the microRNA (miRNA, miR) networks regulated by exercise could contribute to physiological cardiac hypertrophy [5]. miRNAs are a type of small noncoding RNA (18–23 bp) that can cause translational repression or cleavage of mRNAs and posttranscriptional silencing. miRNAs are considered to be promising therapeutic targets for numerous cardiovascular diseases and have been shown to play a key role in physiological hypertrophy by regulating various cellular functions, including decreasing fibrosis and apoptosis and inducing cell growth and angiogenesis [6]. Different studies focused on physiological hypertrophy identified various miRNAs and their potential pathways due to different experimental conditions; however, the most likely mechanism is still unclear [7–14]. In this study, we collected the current published data concerning miRNAs associated with physiological hypertrophy and predicted the most likely biological pathways underlying exercise-induced physiological cardiac hypertrophy using bioinformatics tools.

## 2. Methods

### 2.1. Data Extraction

We searched the PubMed website using the keywords “microRNA,” “exercise,” and “hypertrophy” to identify all of the published microRNAs associated with exercise-induced cardiac hypertrophy that were eligible for further bioinformatics analysis. We collected all the appropriate published articles and extracted all the data needed for further analysis.

### 2.2. Bioinformatics Analysis

The software MirPath v.3 from the DIANA tools website was used to identify potential miRNA target genes and pathways in our study. TargetScan was used in this study to predict the potential target genes and demonstrate the possible relationships among the databases. Pathway analysis was performed to determine the involvement of coexpressed genes in different biological pathways according to the Kyoto Encyclopedia of Genes and Genomes (KEGG). Gene Ontology (GO) analysis was used to investigate the pathways associated with biological processes, cellular components, and specific molecular functions corresponding to the target genes of miRNAs identified by the software TargetScan. In this study, *P* values less than 0.05 (*P* < 0.05) were considered to be statistically significant.

## 3. Results

### 3.1. The Profiles of Identified miRNAs

As Table 1 shows, 8 studies were included in our study. Mouse models were used in 2 studies and rat models were used in another 6 studies to investigate the miRNAs regulated by various types of exercise [7–14]. Swimming, running, jumping, or wheel running were applied in the above studies. A total of 23 miRNAs were found to be associated with exercise-induced physiological cardiac hypertrophy. A total of 12 miRNAs were upregulated after exercise, while 14 miRNAs were downregulated, and 3 miRNAs showed different results in different reports.

### 3.2. KEGG and GO Pathway Analysis

**Figure 1:**
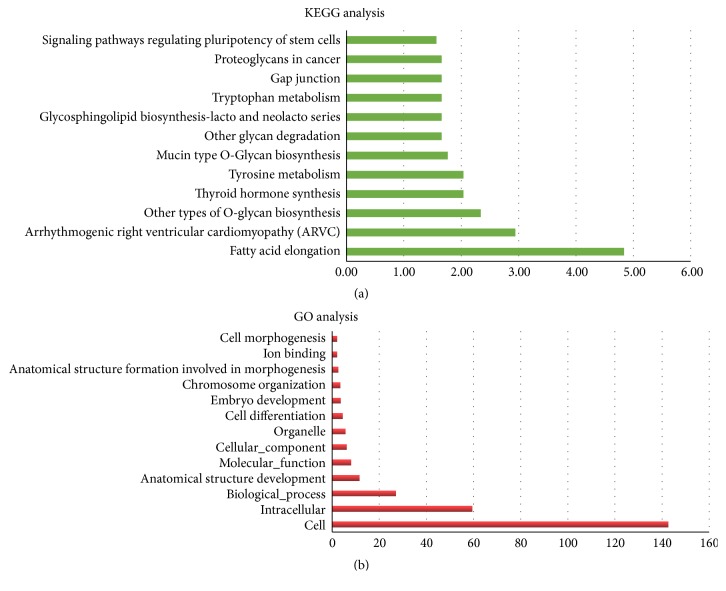
The KEGG and GO pathways incorporated by downregulated microRNAs.

**Figure 2:**
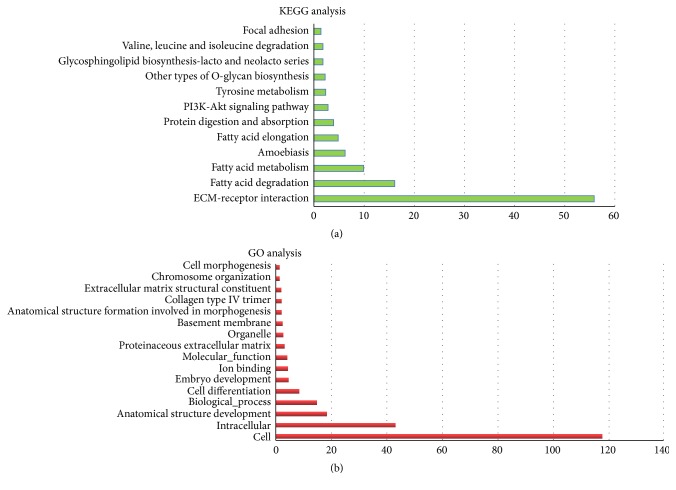
The KEGG and GO pathways incorporated by upregulated microRNAs.

KEGG pathway analysis was used to identify all the potential pathways corresponding to miRNAs regulated by exercise. As shown in Table 2 and Figure 1(a), the significant (*P* < 0.05) pathways corresponding to downregulated miRNAs were as follows: Fatty acid elongation, Arrhythmogenic right ventricular cardiomyopathy (ARVC), Other types of O-glycan biosynthesis, Thyroid hormone synthesis, Tyrosine metabolism, Mucin type O-glycan biosynthesis, Other glycan degradation, Glycosphingolipid biosynthesis-lacto and neolacto series, Tryptophan metabolism, Gap junction, Proteoglycans in cancer, and signalling pathways regulating pluripotency of stem cells. The significant pathways corresponding to upregulated microRNAs were as follows: ECM-receptor interaction, Fatty acid degradation, Fatty acid metabolism, Amoebiasis, Fatty acid elongation, Protein digestion and absorption, PI3 K-Akt signalling, Tyrosine metabolism, Other types of O-glycan biosynthesis, Glycosphingolipid biosynthesis-lacto and neolacto series, Valine, leucine and isoleucine degradation, and Focal adhesion (Table 3 and Figure 2(a)).

Gene Ontology (GO) analysis was used to investigate the pathways associated with biological processes, cellular components, and specific molecular functions corresponding to the target genes of miRNAs identified by the software TargetScan. The pathways corresponding to the target genes of downregulated miRNAs included cell, intracellular biological process, anatomical structure development, molecular function, cellular component, organelle, cell differentiation, embryo development, chromosome organization, anatomical structure formation involved in morphogenesis, ion binding, and cell morphogenesis (Figure 1(b)). Similarly, the following pathways corresponded to the upregulated miRNAs: cell, intracellular, anatomical structure development, biological process, cell differentiation, embryo development, ion binding, molecular function, proteinaceous extracellular matrix, organelle, basement membrane, anatomical structure formation involved in morphogenesis, collagen type IV trimer, extracellular matrix structural constituent, chromosome organization, and cell morphogenesis (Figure 2(b)).

## 4. Discussion

Exercise is widely known as a safe and well-accepted nonpharmacological strategy to improve cardiac function and protect the heart from cardiovascular disorders [15]. It is quite important to find the potential molecular pathways underlying the physiological cardiac hypertrophy induced by exercise. In recent years, an increasing number of studies have focused on miRNAs, which can inhibit the transcription of target mRNAs and regulate various biological pathways [16]. Various studies reveal that miRNAs regulated by exercise are associated with physiological cardiac hypertrophy processes [7, 8]. However, due to the various animal models and the various types of exercise training, different miRNAs and potential biological pathways have been reported in different studies. In several studies, some miRNAs were even reported to be regulated in opposite directions, and thus it is hard to determine which miRNAs and pathways are associated most with exercise-induced hypertrophy [8, 9]. In this study, we collected all the miRNA data from all the published articles about exercise-induced cardiac hypertrophy and investigated the most likely biological pathways using bioinformatics analysis.

The analysis of downregulated miRNAs regulated by exercise revealed that Fatty acid elongation pathway and Arrhythmogenic right ventricular cardiomyopathy (ARVC) pathway could be associated with the hypertrophy process. In the Fatty acid elongation pathway, ELOVL family member 6 (Elovl6) is a microsomal enzyme, which has been shown to regulate the monounsaturated Fatty acids and elongation of C12–16 saturated Fatty acids [17]. Elovl6 is associated with brown adipose tissue (BAT) thermogenic capacity, which could protect against cardiomyocyte injury and suppress cardiac remodelling in catecholamine-induced cardiomyopathy [18]. Our findings first indicate that exercise could contribute to physiological hypertrophy via regulating the Elovl6-BAT pathway. Arrhythmogenic right ventricular cardiomyopathy (ARVC) is typically an autosomal dominant heart muscle disease, which is always accompanied by ventricular enlargement, heart dysfunction, and lethal arrhythmias [19]. ARVC is one of the most common primary reasons of sudden death in young people and athletes. Experimental and clinical studies indicated that exercise might induce ARVC [20]. In this study, we also elucidate the potential association between exercise and ARVC via miRNA regulation.

The analysis of upregulated miRNAs induced by exercise revealed that the ECM-receptor interaction pathway is the most valuable one. It was recently identified that in the mouse model of angiotensin II-induced cardiac remodelling, ECM-receptor interaction pathway may be involved in the process of cardiac remodelling [21, 22]. Here, we also indicate the relationship between this pathway and cardiac hypertrophy using bioinformatics analysis. This association needs to be more clearly elucidated in the future. We speculate that exercise can decrease fibrosis by targeting the ECM-receptor interaction pathway.

In conclusion, we predicted the most likely biological pathways associated with physiological cardiac hypertrophy induced by exercise in this study through extracting published miRNA data and performing analysis using bioinformatics methods, which may help us better understand the regulatory networks of exercise-induced physiological cardiac hypertrophy. However, more in-depth studies and clinical investigations are still needed in the future.

## Figures and Tables

**Table 1:** The microRNAs regulated by exercise.

MicroRNAs	Exercise training	Regulation	Reference
MiR-133a (rno-miR-133a-3p)	Running & swimming	Down	17468766 & 21447748
MiR-1 (rno-miR-1-3p)	Running & swimming	Down	17468766 & 21447748
MiR-27a (rno-miR-27a-3p)	Swimming	Up	21709209
MiR-27b (rno-miR-27b-3p)	Swimming	Up	21709209
MiR-143 (rno-miR-143-3p, mmu-miR-143-3p)	Swimming & wheel running	Down	21709209 & 24751578
miR-26b (mmu-miR-26b-5p)	Wheel running	Down	24751578
miR-150 (mmu-miR-150-5p)	Wheel running	Up	24751578
miRNA-133b (rno-miR-133b-3p)	Swimming	Down	21447748
miRNA-29c (rno-miR-29c-3p)	Swimming	Up	21447748
miRNA-214 (rno-miR-214-3p)	Jumping	Down	25822872
miR-208b (rno-mir-208b)	Swimming	Up	25793527
miR-30e (rno-miR-30e-5p)	Swimming	Up	25793527
miR-19b (rno-miR-19b-3p)	Swimming	Up	25793527
miR-99b (rno-miR-99b-5p)	Swimming	Down	25793527
miR-100 (rno-miR-100-5p)	Swimming	Down	25793527
miR-191a (rno-mir-191a)	Swimming	Down	25793527
miR-22 (rno-miR-22-3p)	Swimming	Down	25793527
miR-181a (rno-miR-181a-5p)	Swimming	Down	25793527
miR-222 (mmu-miR-222-3p)	Swimming & wheel running	Up	25863248
miRNA-21 (rno-miR-21-5p)	Swimming	Up	23812090
miRNA-145 (rno-miR-145-5p)	Swimming	Up	23812090
miRNA-144 (rno-miR-144-3p)	Swimming	Up	23812090
miRNA-124 (rno-miR-124-3p)	Swimming	Down	23812090

**Table 2:** The KEGG pathways incorporated by downregulated miRNAs.

KEGG pathway	−log⁡(*P* value)	Genes
Fatty acid elongation	4.84	Hadha, Elovl6
Arrhythmogenic right ventricular cardiomyopathy (ARVC)	2.94	Cacng4, Cacna2d1, Sgcd, Jup, Itgb8, Gja1
Other types of O-glycan biosynthesis	2.34	B3glct, Pomt2, Fut9, Colgalt2
Thyroid hormone synthesis	2.04	Duox2, Tg
Tyrosine metabolism	2.04	Maoa, Aldh1a3
Mucin type O-Glycan biosynthesis	1.76	Galnt16, Galnt7
Other glycan degradation	1.66	Neu3
Glycosphingolipid biosynthesis-lacto and neolacto series	1.66	B3galt2, Fut9
Tryptophan metabolism	1.66	Afmid, Hadha, Tph2, Maoa, Ogdhl
Gap junction	1.66	Tjp1, Egfr, Nras, Grm5, Gja1
Proteoglycans in cancer	1.66	Wnt2, Cttn, Wnt4, Fgfr1, Egfr, Fn1, Nras, Erbb3, Rock2, Flna, Fzd3, Wnt2b, Pik3cd, Cav3, Ppp1cc
Signaling pathways regulating pluripotency of stem cells	1.57	Fgfr3, Wnt2, Wnt4, Fgfr1, Nras, Nodal, Smad4, Fzd3, Wnt2b, Pik3cd, Acvr1c

**Table 3:** The KEGG pathways incorporated by upregulated miRNAs.

KEGG pathway	−log(*P* value)	Genes
ECM-receptor interaction	55.86	Col2a1, Col3a1, Col6a3, Col1a1, Col4a4, Col5a2, Col5a3, Col4a1, Col4a3, Col11a1, Col4a2, Col5a1
Fatty acid degradation	16.12	Hadha, Ehhadh, Acadm
Fatty acid metabolism	9.93	Hadha, Ehhadh, Acadm
Amoebiasis	6.21	Col2a1, Col3a1, Col1a1, Prkx, Col4a4, Col5a2, Col5a3, Col4a1, Col4a3, Col11a1, Col4a2, Col5a1
Fatty acid elongation	4.85	Hadha
Protein digestion and absorption	3.93	Col2a1, Col3a1, Col6a3, Col1a1, Col4a4, Col5a2, Col5a3, Col9a1, Col4a1, Col4a3, Col11a1, Atp1b4, Col4a2, Col5a1
PI3K-Akt signaling pathway	2.81	Tsc1.Col2a1.Col3a1.Ddit4.Fgf.Col6a3.Lpar6.Fgf12.Eif4b.Col1a1.Irs1.Them4.Vegfa. Egfr.Ppp2ca.Ghr.Col4a.Tek.Col5a2.Osmr.Ppp2cb.Col5a.Ppp2r3c.Gng5.Col4a1. Col4a.Col11a1.Myb.Gnb3.Col4a.Col5a1.Kit
Tyrosine metabolism	2.36	Maoa, ldh1a3
Other types of O-glycan biosynthesis	2.23	Pomt2, Fut9, Colgalt2
Glycosphingolipid biosynthesis-lacto and neolacto series	1.81	B3galt2, Fut9
Valine, leucine and isoleucine degradation	1.81	Hadha, Ehhadh, Acadm
Focal adhesion	1.41	Col2a1.Col3a1.Vav3.Col6a3.Col1a1.Vegfa.Crkl.Egfr.Col4a.Col5a2.Col5a3.Mylk. Col4a1.Col4a3.Col11a1.Col4a2.Col5a1.Ppp1ccsee interacti
